# Use of Microorganisms as Nutritional and Functional Feedstuffs for Nursery Pigs and Broilers

**DOI:** 10.3390/ani12223141

**Published:** 2022-11-14

**Authors:** Yi-Chi Cheng, Sung Woo Kim

**Affiliations:** Department of Animal Science, North Carolina State University, Raleigh, NC 27695, USA

**Keywords:** bacteria, feedstuffs, feed additives, nursery pigs, microalgae, yeasts

## Abstract

**Simple Summary:**

The use of microorganisms has become a trend as nutritional and functional feedstuffs become widely used in swine and poultry diets. Microorganisms, as coproducts obtained from the food industry and biorefineries, can reduce not only the burdens of the natural ecosystem but also the high costs of feedstuffs. It is possible to mitigate food and land competition with humans in the current global issues. These microorganisms could be promising and sustainable alternatives in animal diets because they contain highly valuable proteins, amino acids, fatty acid composition, and biogenic metabolites, which are beneficial for animal production. Microorganisms could be good alternatives to replace plant and animal-based protein supplements with high protein and a balanced amino acid composition. Lipid-rich microalgae and yeasts could be alternative energy feeds with valuable fatty acids used to enhance intestinal health and meat quality. In addition, microorganisms could be functional feed additives due to their cell contents and their cell wall bioactive components. However, there still are some limitations to using microorganisms, including the sources and dose of those microorganisms, which may cause negative effects on growth and health. Thus, this research focused on investigating the use of nutritional and functional microorganisms as feedstuffs and feed additives to replace conventional feedstuffs for enhancing the growth and intestinal health of nursery pigs and broilers.

**Abstract:**

The objectives of this review paper are to introduce the structures and composition of various microorganisms, to show some applications of single cells as alternative protein supplements or energy feeds in swine and poultry diets, and to discuss the functional effects of microorganisms as feed additives on the growth performance and intestinal health of nursery pigs and broilers. Microorganisms, including bacteria, yeasts, and microalgae, have been commonly supplemented in animal diets because they are cost-effective, stable, and have quantitative production that provides nutritional and functional benefits to pigs and broilers. Microorganisms could be alternative antibiotics to enhance intestinal health due to bioactive components from cell wall components, which interact with receptors on epithelial and immune cells. In addition, bioactive components could be digested by intestinal microbiota to produce short-chain fatty acids and enhance energy utilization. Otherwise, microorganisms such as single-cell protein (SCP) and single-cell oils (SCOs) are sustainable and economic choices to replace conventional protein supplements and energy feeds. Supplementing microorganisms as feedstuffs and feed additives improved the average daily gain by 1.83%, the daily feed intake by 0.24%, and the feed efficiency by 1.46% in pigs and broilers. Based on the properties of each microorganism, traditional protein supplements, energy feeds, and functional feed additives could be replaced by microorganisms, which have shown benefits to animal’s growth and health. Therefore, specific microorganisms could be promising alternatives as nutritional and functional feedstuffs in animal diets.

## 1. Introduction

Animal diets make up 70% of the total costs of animal production [1]. Soybean meal (SBM) and corn are the main protein supplement and energy feed in animal diets, respectively. However, plant protein supplements contain anti-nutritional factors, including trypsin inhibitors, flatulence-producing compounds, and allergenic proteins, which restrict growth performance and intestinal development [2,3]. Some anti-nutritional factors could be eliminated via fermentation by using yeasts or bacteria to enhance nutrient bioavailability [4]. After fermentation, these microorganisms could be supplemented as coproducts in animal diets due to their valuable amino acids, vitamins, minerals, nucleotides, enzymes, and other metabolites [5,6,7]. In addition, the use of plant-based feedstuffs is dependent on seasonal availability and is limited to land use [8], whereas the use of microorganisms has fewer availability concerns and could be produced on a large scale in less time.

Animal-based feedstuffs are commonly supplemented in nursery diets to enhance growth performance, nutrient digestibility, and intestinal health [3,9,10]. Although animal-based feedstuffs have positive effects on growth and health development in pigs and broilers, these feedstuffs are expensive and in short supply [11,12]. It is important for nutritionists to seek alternative feedstuffs so that animal producers can reduce the cost burden while maintaining the growth performance of pigs and broilers. For alternative feedstuffs in pig and poultry diets, some key points need to be considered, including nutritional values, availability, palatability, and consistency [13,14]. Among alternative feedstuffs, coproducts from the food industry, insects, and some microorganisms can replace expensive feedstuffs in animal diets.

Coproducts from the food industry are convenient and easily available for delivery to feed mills. In the research from Kwak and Kang [15] using finishing pigs, a food waste mixture (70% food waste, 10% poultry litter, and 13% bakery coproducts) with an aerobic microbial culture could be supplemented in diets, replacing corn and SBM, without adverse effects on their growth and meat quality. However, supplementing bakery meal as an alternative energy feed reduces growth performance and the digestibility of AA in diets fed to nursery and growing pigs [16,17,18]. Candy coproducts could partially replace whey permeate without negative effects on the growth performance of nursery pigs [19]. The concern of using food coproducts is the variable nutrient composition by different processes and sources; therefore, it is important to analyze nutrient composition before formulation. Insect meal contains high protein and lipid content and is used to replace SBM and animal-based protein supplements [20,21]. Even though some studies demonstrated that corn–insect diets had better growth performance than corn–SBM diets in poultry diets, the price of insect meal remains high due to the low production [21,22]. Some microorganisms have been commonly supplemented in animal diets because they are cost-effective, stable, and their quantitative production provides nutritional and functional benefits to pigs and broilers. In addition, specific microorganisms could be divided into groups with different characteristics and functions. Further details are reviewed in the following sections.

### 1.1. Bacteria

**Figure 1:**
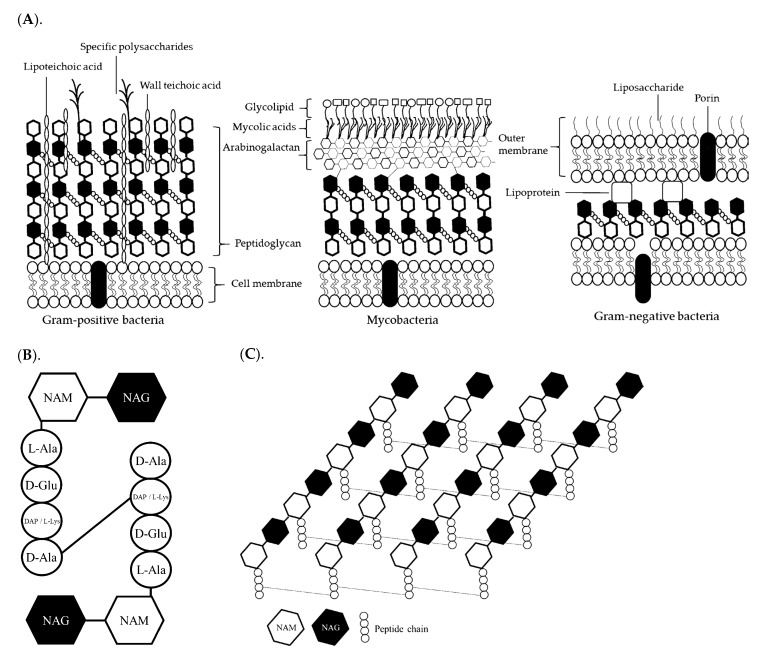
The structure of Gram-positive and Gram-negative bacterial cell walls (**A**), peptidoglycan (PG) structure (**B**), and the cross-linking of PG chains in bacteria (**C**). Concepts were based on Koch (2006) [37]; Kang et al. (2016) [38]; Pazos and Peters (2019) [25]. LPS, lipopolysaccharides; NAM, N-acetylmuramic acid; NAG, N-acetylglucosamine; L-Ala, L-alanine; D-Glu, D-glutamic acid; DAP, Diaminopimelic acid; L-Lys, L-lysine; D-Ala, D-Alanine.

Bacteria are unicellular and relatively small with a size range of 0.5 to 5.0 µm [23]. Bacteria are rich in lipids, proteins, and amino acids (Table 1). In addition, bacteria are categorized into two groups, Gram-positive and Gram-negative, based on different cell wall structures (Figure 1A). Peptidoglycan (PGN) is the major component (40 to 60%) of the cell wall and is made of N-acetylglucosamine (NAG), N-acetylmuramic acid (NAM), and short peptide chains, including L-alanine, D-glutamic acid, either L-lysine or diaminopimelic acid (DAP), and D-alanine [24,25] (Figure 1B). In the cell wall, cross-linking of the PGN envelope enhances the strength of the structure (Figure 1C). Gram-positive bacteria have a thicker cell wall due to more PGN envelopes in the cell wall than Gram-negative bacteria [26], whereas Gram-negative bacteria have an outer membrane, called the lipopolysaccharide (LPS), which may be toxic and affect animal health [23,27]. Based on properties of bacterial cells, they were commonly used to replace fish meal in aquacultural diets without negative effects on intestinal health and growth [28]. Some studies demonstrated that the thick cell wall is non-digestible in mono-gastric animals; however, some bacterial cell walls can be utilized by the intestinal microbiota to enhance intestinal health [27,29].

**Table 1:** Characteristics and nutrient content of bacteria, yeasts, and microalgae.

	Bacteria		Yeast	Microalgae
DM ^1^, %	90 to 95		93	94
CP, % DM basis	50 to 80		12 to 53	10 to 70
Lipid, % DM basis	7 to 15		1 to 40	3 to 71
Total Fiber, % DM basis	3 to 6		2 to 40	10 to 66
Cell wall contents	Gram + ^2^	Gram − ^2^	Mannoprotein (35 to 40%); 1,3 β-glucan (50 to 55%); 1,6 β-glucan (5 to 10%); Chitin (up to 3%)	Polysaccharide (1 to 12%) Soluble protein (up to 4.5%)
	20 to 80 nm; PN ^3^ (40 to 60%); Teichoic acid (up to 40%); Arabinogalactan (10 to 20%)	8 to 10 nm; PN (10 to 20%); LPS ^4^; Lipoprotein		
References	[30,31]		[23,32,33]	[34,35,36]

^1^ DM—dry matter. ^2^ Gram +/−—Gram-positive (+)/negative (−) bacteria. ^3^ PN—peptidoglycan. ^4^ LPS—lipopolysaccharide.

### 1.2. Yeasts

**Figure 2:**
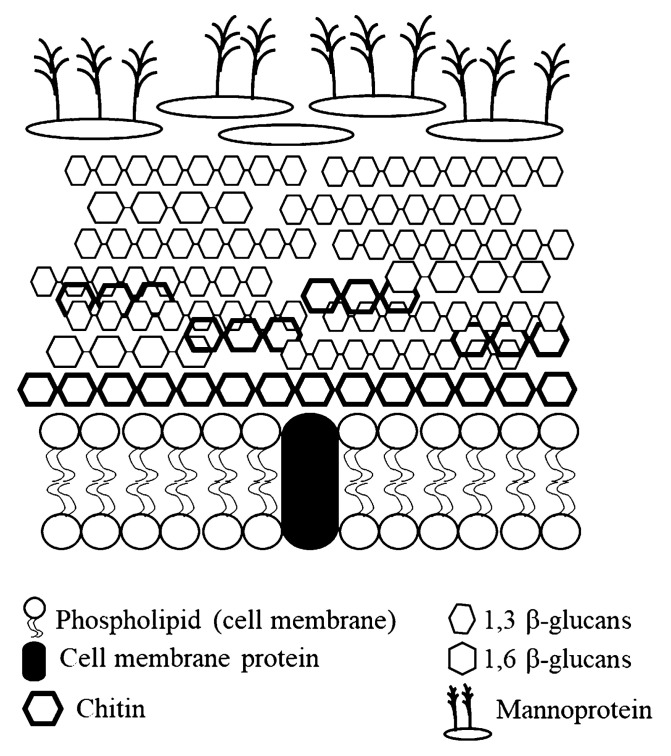
The structure of yeast cell wall. Concepts were based on Lipke and Ovalle (1998) [45]; Kogan et al. (2008) [46]; Morphology (2012) [32].

Yeast sizes are variable from 2 to 50 μm in length and 1 to 10 μm in width [32]. Yeasts could grow under aerobic respiration, so they are generally used for brewing to produce alcohol [39]. In both inner and outer cell walls (Figure 2), β-glucans and mannoprotein are the major components [23] (Table 1). Chitin is a minor component, contributing approximately 1 to 3% in the yeast cell wall, and β-1,6 glucan links to the inner and outer walls, strengthening the cell structure [40]. Interestingly, yeast cell walls could stimulate animals to secrete protease and glucanase to release cell contents and cause fragmentation of cell walls [41]. Some studies demonstrated that β-glucans and mannoprotein from yeasts can enhance growth performance and intestinal health in pigs [42,43,44].

### 1.3. Microalgae

**Figure 3:**
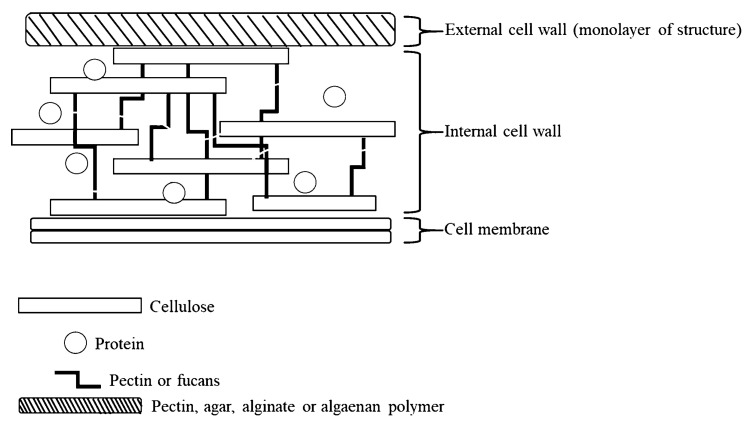
The structure of microalgae cell wall. Concepts were based on Velazquez-Lucio et al. (2018) [54]; Pôjo et al. (2021) [55].

Within the microorganisms, microalgae are rich in essential fatty acids (FA), vitamins, and minerals [34] (Table 1). Cell wall components of microalgae are primarily made of cellulose, with pectin, fucan, xylan, and mannan as minor components [47,48] (Figure 3). Pyrrophyta contains two flagella and chlorophyll with carotenoid and xanthophyll as bioactive components, which accumulate starch via photosynthesis [49]. Compared to other types of microalgae, Chrysophyta includes cellulose, silica, and calcium carbonate in the cell wall and are able to accumulate lipids, including omega-3 [50], therefore, they are generally used as energy feeds [51,52,53].

There are several advantages of using nutritional and functional microorganisms: (1) Microbial production is an applicable and economical technology to obtain stable products and maintain the cell culture [56,57]. (2) It is eco-friendly because microorganisms that are considered as coproducts from the food industry or biofuel production could be recyclable and supplemented in animal diets [56,58]. (3) Microorganisms are high in nutrients, such as protein, AA, fats, and vitamins, and can be useful in animal diets. However, there are some issues with using microorganisms in animal diets due to low digestibility, heavy metals, and toxicity [12]. Although the cell wall is non-digestible in pigs, bioactive components could be extracted to enhance intestinal health in pigs and broilers [59,60,61].

The hypothesis of this review is that supplementing microorganisms as nutritional and functional feed additives in nursery diets is feasible. To achieve this, the objectives are as follows: (i) To introduce the structures and composition of various microorganisms, (ii) to show some applications of microorganisms as alternative protein supplements or energy feeds in animal diets, and (iii) to discuss the functional effects of microorganisms as feed additives on the growth performance and intestinal health of pigs and broilers.

## 2. Microorganisms as Functional Feed Additives

### 2.1. Introduction of Functional Feed Additives

The intestinal microbiota is an indicator of intestinal health, which assists digestion and absorption of nutrients, the development of the intestinal immune system, and the inhibition of the colonization of harmful microbiota [62,63,64]. Young animals are susceptible to pathogenic infections due to their immature gastrointestinal (GI) tract and microbiota community. Therefore, diet composition plays a critical role in developing the balance of intestinal microbiota [65,66]. Antibiotics have been supplemented in young animal diets to avoid disease and enhance the growth rate [67]. The role of antibiotics is to inhibit pathogen replication and destroy cell wall synthesis [68]. However, the use of antibiotics gives rise to pathogens developing antibiotic resistance and affecting the intestinal microbial population [69]. Many countries have banned the use of antibiotics due to chemical residues in animals and antibiotic resistance transferred to humans [68]. Consequently, alternative antibiotics, including prebiotics, probiotics, or postbiotics derived from bacteria, yeast, and microalgae, can be a safer alternative for use in swine production [29,42,43]. Probiotics are live microorganisms, which benefit animal growth and the intestinal microbial community [27,70]. Prebiotics are polysaccharides obtained from the cell walls of microorganisms [35]. On the other hand, postbiotics are metabolites and cell contents extracted from probiotics [71]. Probiotics, prebiotics, and postbiotics not only balance intestinal microbiota diversity [27,42] but also have positive effects on the immune system by preventing intestinal inflammation [29,43].

### 2.2. Mechanism and Application

#### 2.2.1. Bacteria

The mechanism of immune response is complicated and varies based on different types of bacteria. Among the bacterial cell walls, PGN, teichoic acid (TA), and S-layers are the main cell wall components in most Gram-positive bacteria, whereas *Mycobacteria* contain PGN, arabinogalactans (AG), and mycolic acids, which make up the top layer [30]. Different from the Gram-positive bacteria, Gram-negative bacteria contain less peptidoglycan, porin, and lipopolysaccharides (LPS) in the cell wall. Peptidoglycans, TA, and AG interact with receptors on the epithelial and immune cells, including Toll-like receptor 2 (TLR2) and a cluster of differentiation 14 (CD14), which is a co-receptor of TLR2, and on the intestinal epithelial cells (IECs) [71,72,73]. Peptidoglycans are cleaved by PGN hydrolases into small fragments and recognized by nucleotide binding and oligomerization domain proteins (NOD). The NOD inhibits nuclear factor-κB (NF-κB) and proinflammatory cytokines, interleukin (IL) 6, IL-8, and tumor necrosis factor-alpha (TNF-α) [74,75]. Different from PGN, AG binds to C-type lectin receptor (CLR) on the surface of immune cells, dendritic cells, and macrophages that decrease the activity of NF-κB and inhibit the release of proinflammatory cytokines [76].

When animals are infected by LPS from pathogenic bacteria, LPS stimulates TLR4 and NF-κB releasing interferons and pro-inflammatory cytokines. Probiotics, prebiotics, and postbiotics could act as immunostimulators to stimulate TLR to inhibit NF-κB and activate an anti-inflammatory response [77,78,79]. For example, selected *Bacillus* sp. not only help to decrease diarrhea caused by *Escherichia coli* (*E. coli*) infection but also improve growth performance [27,80] (Table 2). However, it is observed that not all *Bacillus* sp. exhibit positive effects on growth performance [81]. In addition, Lactic acid bacteria (LAB), including *Enterococcus* sp. and *Lactobacillus* sp., commonly occur in the GI tract due to a favorable environment and strong adhesion to the intestinal epithelial cells [82], which stimulate the inflammatory response to release cytokines and chemokines as pathogens enter the body [79]. This can increase lymphocyte proliferation and macrophage phagocytosis to reduce aggregated pathogens in the intestine [82]. Taras et al. [83] demonstrated that *Enterococcus faecium* fed to sows can reduce the mortality of newborn piglets and post-weaning diarrhea. *Enterococcus faecium* reduced serum IgG [67] and tended to reduce chlamydial infection in newborn piglets from infected sows [84]. Therefore, bacteria and their bioactive components could be functional feed additives for beneficial immune responses in nursery pigs.

#### 2.2.2. Yeasts

Beta-glucans, mannoprotein, and chitin are the main cell wall components of yeasts. Many studies have investigated how the yeast cell wall and its metabolites could modulate immune responses through pattern recognition receptors (PRR), including TLR and CLR [94,95]. Beta-glucans derived from yeast cell walls bind to the TLR2 and CLR family and dectin-1 receptor on enterocytes and immune cells [96,97]. Activated receptors give rise to Ig secretion and increase the number of goblet cells for the maintenance of intestinal structural integrity [98]. Li et al. (2006) [99] demonstrated that pigs fed β-glucans can inhibit secretion of TNF-α and IL-6 due to increased IL-10; therefore, nutrients would be utilized for increased growth performance rather than for immune responses. Moreover, β-glucans can increase the number of LAB, which improve intestinal health and alleviate pathogens infected [100].

Mannoprotein, located on the external cell wall, contains oligomannoside chains to produce mannose oligosaccharides in yeasts [46]. Mannose has the ability to bind to the mannose-specific lectin-type receptors on pathogenic bacteria or viruses to prevent colonization of the intestinal villi [60]. Additionally, mannose also binds to TLR4 and dectin-2 receptors to activate the immune responses releasing anti-inflammatory cytokines to avoid inflammation [96,97]. Yeasts provided to sows during the gestation and lactation period could improve the growth performance of offspring [101,102,103] because of positive effects on the establishment of beneficial microbiota in the GI tract [104]. Therefore, nursery pigs continuously fed yeasts in their diets showed enhanced digestibility of nutrients [43,104] and had positive effects on intestinal health and morphology by β-glucans, which can enhance growth performance [43] (Table 3). Although the high dose of β-glucans may reduce growth performance in pigs [99], yeasts and their metabolites may have functions similar to antibiotics to enhance growth and reduce inflammation [105].

#### 2.2.3. Microalgae

The bioactive components of microalgae are variable across different species. Many microalgae contain high omega-3 FA, which is converted to polyunsaturated fatty acids (PUFA), eicosapentaenoic acid (EPA), and docosahexaenoic acid (DHA) [114]. Both PUFAs are beneficial to the integrity of cell membranes and reduce inflammation and oxidation [115]. In addition, the use of DHA-rich microalgae could improve meat quality by changing FA composition and reducing the backfat thickness [116,117]. Kibria and Kim (2019) [113] demonstrated that nursery pigs fed *Schizochytrium* sp. developed an enhanced immune system and displayed improved nutrient digestibility and feed efficiency (Table 3). Beta-carotene, one type of carotenoid produced by *Schizochytrium* sp. and the family *Chrysophyceae*, could be antioxidants and immunomodulators [118,119]. In addition, the flavonoid is another common bioactive component, which has anti-inflammatory effects to inhibit NOD-, LRR-, and pyrin domain-containing protein 3 releasing pro-inflammatory cytokines [120]. Furbeyre et al. (2017) [65] demonstrated that pigs fed *Chlorella* and *Arthrospira platensis* had reduced incidence of diarrhea and provided antibiotic function to maintain the intestinal morphology in newly nursery pigs, which may increase beneficial microbiota in the intestine and nutrient digestibility, respectively.

Some microorganisms have been used as functional feed additives with clear mechanisms in immune responses. Their functional cell wall components, including peptidoglycan, teichoic acid, β-glucans, mannoprotein oligosaccharides, and flavonoids, have positive effects on animal’s intestinal health to increase growth and nutrient digestibility. In addition, yeasts and microalgae have prebiotic effects on polysaccharides from the cell wall. Polysaccharides could be digested by intestinal microbiota to produce short-chain fatty acids (SCFA) and enhance energy utilization [35,121]. Short-chain fatty acids bind and activate G protein-coupled receptors related to lipid and glucose metabolism [31] that increase fatty acid oxidation in muscle and reduce fat deposition in adipose tissue [122]. Shen et al. (2009) [43] reported that increased SCFA production may be correlated with improved marbling scores. A recent study demonstrated that SCFA infusion in the ileum increased dressing weight and improved carcass traits by reducing N excretion and regulating lipid metabolism in growing to finishing pigs [123]. However, there are some concerns surrounding the use of microorganisms that may contain biogenic toxins, such as purines and heavy metals [124]. New technology could reduce these toxins and break the cell wall of microalgae to release functional metabolites as valuable feed additives in the future.

## 3. Single Cell Protein (SCP)

### 3.1. Introduction of SCP

Conventional protein supplements, soybean meal (SBM), and animal-based protein supplements, including meat and bone meal, blood plasma, and fish meal, are mainly utilized in animal diets. Soybean meal has anti-nutritional factors, including the trypsin inhibitor, glycinin, and flatulence-producing oligosaccharides, which reduce nutrient digestibility in diets fed to pigs [2,3,125]. Although animal-based protein supplements are highly digestible and can improve health and growth in pigs and broilers [126], they are relatively expensive and in short supply [11,127]. Therefore, SCP is the sustainable and economic choice to replace conventional protein supplements.

Single-cell proteins not only contain high protein content in the cell, namely, 30 to 50% in yeast, 50 to 80% in bacteria, and 60 to 70% in microalgae, but can also be efficiently produced [128]. The SCP has highly valuable protein and AAs, similar to SBM and animal protein supplements [12,129] (Table 4). Furthermore, the use of microorganisms is more eco-friendly and can reduce land usage and carbon production [130]. Many studies have demonstrated that SCP could be a beneficial alternative protein supplement to animals, reducing the portion of conventional protein supplements in diets to enhance growth performance [131,132], animal health [133,134], and meat quality [135,136].

### 3.2. Application of SCP

#### 3.2.1. Bacteria

Based on the nutrient composition, bacterial protein supplements provide a similar amino acid composition to SBM and fish meal [138,144]. There are some bacterial protein supplements used in pig and poultry diets, including *Corynebacterium glutamicum* [138,139], *Methylobacterium extorquens* [145], and *Methylococcus capsulatus* [135,146] (Table 4). Supplementing *Methylococcus capsulate* up to 12 % to replace SBM and fish meal in nursery diets may improve growth performance and meat quality due to changes in FA composition in meat [131,135]. In broiler diets, supplementing *Methylophilus methylotrophus* negatively affected growth performance, whereas intestinal health, microbial community, and disease resistance were improved [147,148,149].

*Corynebacterium glutamicum* and *E. coli* are mainly used to produce AA [150,151]. These bacteria have been considered waste after AA production, but they contain high levels of protein and AA [139]. *Escherichia coli* is Gram-negative bacteria with double layers of membrane and contains LPS. Some strains of *E. coli* may cause low nutrient digestibility and inflammation by binding to TLR4 on enterocytes and activating inflammatory effects [96,152]. However, *Corynebacterium glutamicum* is a Gram-positive and endotoxin-free bacterium, which is generally recognized as safe. This bacterium could be considered a single-cell protein supplemented in nursery diets that can improve growth performance and stimulate immune responses by increasing immunoglobulins (Ig) due to the bioactive components from the CGCM cell wall [139]. Within the same genus, *Corynebacterium ammoniagenes* supplemented up to 1% replacing SBM in poultry diets enhanced the daily gain and feed efficiency; however, increasing the inclusion of *Corynebacterium ammoniagenes* caused negative effects on growth and meat quality as a result of the low digestibility of protein and AA [132,153]. The overall result of supplementing bacterial protein supplements improved ADG by 0.12%, while reducing ADFI by 0.7% and FE by 0.41% in pigs and broilers (Table 5).

#### 3.2.2. Yeasts

Torula yeast (*Candida utilis*) and brewer’s yeast (*Saccharomyces cerevisiae*) contain 45 to 55% protein in the total cell, which could be considered protein supplements in animal diets [155,156,157,158] (Table 6). Yeasts are rich in Lys but insufficient in sulfur AA, therefore additional Met must be considered in feed formulation [155]. Yeasts would be utilized by intestinal microbiota and produce SCFA, which are energy feeds for the health of intestinal epithelial cells [159]. The overall impact of supplementing yeast protein supplements may reduce ADG by 0.16% and ADFI by 1.86% but improve FE by 1.89% in pigs and broilers (Table 7). However, studies demonstrated that supplementing yeasts at a certain level and replacing conventional protein supplements had positive effects on growth performance, nutrient digestibility, and intestinal morphology in nursery pigs [156,160,161,162]. The reason for enhanced growth performance is due to β-glucans and mannoprotein from the cell wall, which are beneficial to animal’s health [41,158]. However, some studies demonstrated that supplementing yeasts and replacing SBM and fish meal in the diets did not affect intestinal health in nursery pigs regarding the immune response and liver biomarkers [66,159].

#### 3.2.3. Microalgae

Microalgae contain high values of oil and increased protein concentrations after oil extraction [171,172] (Table 6). Therefore, the de-fatted microalgae could be used as protein supplements, and their protein contents vary from 12 to 65% CP based on different microalgae [173]. The overall impact of supplementing microalgal protein supplements improves ADG by 2.24% and FE by 0.44% but reduces ADFI by 0.13% in pigs and broilers (Table 8). Dietary *Arthrospira platensis* of 15%, replacing SBM, is beneficial to growth and health due to enhanced activities of digestive enzymes and nutrient utilization [174]. This microalga could improve meat quality by changing the FA composition and increasing flavor, while the color of meat is more yellow due to the high amount of zeaxanthin in the microalgae [174,175]. However, the growth performance in pigs fed *Arthrospira platensis* did not change, whereas improvements were seen in regard to oxidative stress in muscles and meat quality [176,177]. In contrast, the use of microorganisms *Desmodesmus* sp. and *Nannochloropsis oceanica* in pig and broiler diets promoted protein and FA synthesis by increased expression of the mammalian target of rapamycin (mTOR) and acetyl CoA carboxylase [134,178,179].

The usage levels of microorganisms should be approached cautiously, as increasing the dose may cause negative effects on growth and health in animals due to the low digestibility of the cell wall of microorganisms and over-reaction to bioactive components [139]. The thick cell wall could be broken down by various technologies, including autolysis and hydrolysis [181,182]. Each technique may reduce palatability and growth performance in animals due to the change in nutrient composition [181,183] and affect nutritional values [7].

In summary, microorganisms such as SCPs are beneficial to animals’ growth, health, and meat quality. However, SCP may cause some problems with the usage levels and technology of production [184,185], which need to be considered during feed formulation. Therefore, further studies are needed to discuss the appropriate use of single-cell proteins in animals.

## 4. Single-Cell Oil (SCO)

### 4.1. Introduction of SCO

Dietary lipids provide critical energy in feeds and essential fatty acids (EFA), increase nutrient absorption, and reduce feed dust [137]. For energy feeds, vegetable oils and animal fats have been used for over 35 years around the world [186]. Common lipid sources in pig diets are vegetable oils, animal fats, and animal–vegetable fat blends. Fats of animal origin, including poultry fat, tallow, and lard, have been used for a long time due to their higher digestibility in pigs [187,188]. The production of animal fats has increased in recent years and supplied the food industry, animal industry, and diesel production with approximately 3000 million pounds in 2019, but the price also increased from 20 cents/pound to 30 cents/pound from 2006 to 2019 in the U.S [189]. In addition, the European Union has become concerned with the use of animal fat regarding animal health due to disease, bovine spongiform encephalopathy, and chemical contaminations [190]. Vegetable oils, including soybean oil, corn oil, palm oil, and coconut oil, supplemented in diets may enhance higher amounts of long-chain n-3 poly-unsaturated fatty acids (PUFA) in carcasses [191,192]. Although vegetable oils are popularly utilized in various areas including the biodiesel and food industries, the production of vegetable oils competes for land with humans and emits thousands of tons of CO_2_ [193]. Therefore, the rise in costs of conventional oils as energy feeds may be substituted with SCOs, such as *Lipomyces starkeyi*, *Yarrowia lipolytica*, and *Schizochytrium* species [170,194,195].

The advantages of SCO include decreased land usage, lower cost, and a shorter life cycle for large-scale production. Apart from their high protein content, SCO also has valuable fatty acids (Table 9). Microbial oils contain over 20% of lipid content and valuable polyunsaturated fatty acids (PUFA), which enhance immunity in young animals [196,197]. Fatty acid composition from different dietary lipids may affect animal growth performance [198], energy digestibility [198,199,200], intestinal health [201], and meat quality [202,203]. In fish diets, fish need high n-3 PUFA, including EPA and DHA, so plant oils and fish oils are the main energy feeds for fish [204]. Some studies demonstrated that SCOs replacing conventional lipids in diets can be practically used in aquaculture due to similar fatty acid composition and no adverse effects on fish growth and quality [205,206,207].

### 4.2. Application of SCO

Within SCOs, oleaginous yeasts have been involved in various biotechnological applications [210,211] (Table 10). *Yarrowia lipolytica* has the ability to produce valuable protein, lipids, lipolytic enzymes, and organic acids, which have been widely used in the food industry [212]. *Yarrowia lipolytica* is not only an alternative protein supplement in animal diets but also a lipid source containing 20% lipids in the cell [206]. When 3% dried *Yarrowia lipolytica* was used as a protein supplement, replacing soybean meal, it improved ADG and feed efficiency [165]. However, 6% *Yarrowia lipolytica,* with its high lipid content, resulted in diarrhea in piglets, as well as a reduction in the growth performance of nursery pigs [165]. In addition, Cheng et al. (2022) [213] demonstrated that 1.5% *Yarrowia lipolytica* used as energy feeds, replacing poultry fat, maintained intestinal health and growth performance in nursery pigs, while the thick cell wall may reduce nutrient digestibility when supplementing 3% *Yarrowia lipolytica*. Hatlen et al. (2012) [206] reported that 10 to 30% *Yarrowia lipolytica* supplemented in fish diets improved feed efficiency and protein and energy retention; however, protein digestibility and energy digestibility were reduced due to the indigestible yeast cell wall. The result may indicate that the lysis of yeast cell walls may be required to release nutrients and increase nutrient digestibility in diets. Berge et al. (2013) [214] reported that disrupted *Yarrowia lipolytica* released more lipids from the cell and improved nutrient digestibility. Another oleaginous yeast, *Lipomyces starkeyi*, is a feasible replacement for vegetable oils in fish without adverse effects on fish growth and meat quality [205].

Microalgae is high in n-3 PUFA, especially EPA (20:5n-3) and DHA (22:6n-3), so it is effective for use in young fish [219]. Harel et al. (2002) [220] reported that adding microalgae *Crypthecodinium* sp. to replace fish oil in aquacultural diets demonstrated similar growth performance compared with a commercial control diet. Due to the high arachidonic acid proportion in microalgae, *Crypthecodinium* sp. improved the hatching rate of eggs [220] and reduced mortality during the larval stage of fish [207,221]. Supplementing *Schizochytrium* sp. not only improved growth performance but also enhanced intestinal health in nursery pigs and meat quality in growing to finishing pigs based on functional FA [116,216]. However, microalgae as energy feed are not competitive compared to other sources of oils due to the price of production and animal feasibility and acceptability [14]. Furthermore, microalgae can accumulate heavy metals, which may cause animal health problems, so it should be used cautiously to prevent toxic effects [173,194]. Even though SCOs are not as common as animal fats and plant oils supplemented in pig and broiler diets, SCOs may be promising alternative energy feeds based on their valuable FA for animal health and growth.

## 5. Conclusions

The production of selected microorganisms from fermentation is one of the sustainable solutions for the environmental challenges of animal agriculture. Selected microorganisms with nutritional and functional roles in improving the growth and health of young animals provide enhanced production efficiency and profits in animal agriculture. From the review, the use of selected microorganisms as feedstuffs and feed additives enhanced growth by 1.83%, feed intake by 0.24%, and feed efficiency by 1.46% in nursery pigs and broilers. Selected microorganisms, based on their properties, can reduce the use of traditional protein supplements, energy feeds, and functional feed additives. Collectively, selected microorganisms can be promising alternatives as nutritional and functional feedstuffs in diets for nursery pigs and broilers.

## Figures and Tables

**Table 2:** Examples of bacteria as functional feed additives.

Micro-organism	Species	Feedstuff	Product	Level, %	Improvement, %			Animal Model	Reference
					ADG	ADFI	FE		
Bacteria	*Bacillus* sp.	probiotic	1.0 × 10^10^ CFU/g	0.01	11.1	2.94	8.28	Nursery pigs	[80]
Bacteria	*Bacillus* sp.	probiotic	1.0 × 10^9^ CFU/kg	0.05	18.83	20.5	−0.91	Nursery pigs	[81]
				0.05	2.44	−0.80	1.21		
Bacteria	*Bacillus* sp.	probiotic	3.2 × 10^9^ CFU/kg	0.04	2.65	0.81	1.94	Growing-finishing pigs	[85]
Bacteria	*Bacillus* sp.	probiotic	6.0 × 10^8^ CFU/g	0.05	9.80	−0.44	10.4	Growing pigs	[86]
				0.03	4.93	−0.15	5.11		
Bacteria	*Bacillus* sp.	probiotic	2.4 × 10^8^ CFU/g	0.45	9.12	4.87	4.17	Nursery pigs	[87]
				0.30	6.92	4.06	2.78		
				0.15	2.20	1.01	1.24		
Bacteria	*Bacillus* sp.	probiotic	3.2 × 10^9^ CFU/kg	0.04	2.41	−10.6	6.63	Nursery pigs	[88]
Bacteria	*Clostridium butyricum*	probiotic	1.0 × 10^9^ CFU/kg	0.05	2.54	1.76	0.00	Broilers	[89]
Bacteria	*Enterococcus faecium*	probiotic	2.0 × 10^9^ CFU/kg	0.05	4.93	5.14	0.00	Broilers	[89]
Bacteria	*Enterococcus faecium*	probiotic	2.0 × 10^9^ CFU/kg	0.01	5.50	−0.77	6.01	Nursery pigs	[90]
Bacteria	*Lactobacillus* sp.	postbiotic	6.0 × 10^10^ CFU/g and medium	0.20	26.1	20.0	9.52	Nursery pigs	[91]
Bacteria	*Lactobacillus* sp.	probiotic	1.0 × 10^10^ CFU/g	0.50	4.56	36.2	1.10	Nursery pigs	[88]
Bacteria	*Lactobacillus* sp.	probiotic	5.0 × 10^9^ CFU/kg	0.10	6.06	−7.20	12.5	Nursery pigs	[92]
				0.15	3.56	−7.12	10.3		
				0.20	−1.89	−10.2	8.44		
Bacteria	*Lactobacillus* sp.	probiotic	2.4 × 10^5^ CFU/g	0.10	16.0	10.5	5.16	Nursery pigs	[93]
				0.50	1.91	0.84	1.03		
				0.75	3.35	1.75	1.63		
				1.00	4.16	1.16	2.90		

**Table 3:** Examples of yeasts and microalgae as functional feed additives.

Microorganism	Species	Feedstuff	Product	Level, %	Improvement, %			Animal Model	Reference
					ADG	ADFI	FE		
Yeast	*Saccharomyces cerevisiae*	Postbiotic	Glucans extracted	0.05	10.6	5.16	5.38	Broilers	[106]
				0.10	18.7	13.3	4.93		
				0.15	10.5	10.0	1.35		
				0.20	10.6	4.55	10.8		
Yeast	*Saccharomyces cerevisiae*	Prebiotic	Cell wall extract	0.30	4.71	−0.26	6.25	Broilers	[107]
Yeast	*Saccharomyces cerevisiae*	Prebiotic	Cell wall extract	0.03	−15.7	0.85	5.88	Nursery pigs	[108]
Yeast	*Saccharomyces cerevisiae*	Probiotic	1.0 × 10^9^ CFU/g	0.10	4.48	20.1	−15.4	Nursery pigs	[109]
Yeast	*Saccharomyces cerevisiae*	Postbiotic	yeast culture	0.50	6.31	9.48	0.00	Nursery pigs	[43]
				1.00	−6.80	−1.07	−2.79		
				2.00	−8.01	−5.47	0.93		
Microalgae	*Arthrospira platensis*	Postbiotic	Spray dried algae, Setalg (Pleubian, France)	1.00	−0.25	4.21	−3.28	Nursery pigs	[65]
Microalgae	*Arthrospira platensis*	Postbiotic	Spirulina powder, NeoEnBiz Co. (Bucheon, Republic of Korea)	0.25	1.79	1.12	0.67	Broilers	[110]
				0.50	1.91	0.84	1.03		
				0.75	3.35	1.75	1.63		
				1.00	4.16	1.16	2.90		
Microalgae	*Aurantiochytrium limacinum*	Postbiotic	ALL-G Rich, Alltech Inc. (Lexingtong, KE, USA)	1.00	1.12	−0.38	1.22	Finishing pigs	[111]
Microalgae	*Chlorella* sp.	Postbiotic	Spray dried algae, Setalg	1.00	0.51	−1.40	1.64	Nursery pigs	[65]
Microalgae	*Haematococcus pluvialis*	Postbiotic	Novasta, AstaCarotene AB (Stockholm, Sweden)	0.04	−2.00	−2.20	0.00	Broilers	[112]
				0.18	−1.77	−3.64	1.90		
				0.90	−0.74	−2.43	1.27		
Microalgae	*Schizochytrium* sp.	Postbiotic	JB5, JINIS Co., Ltd. (Jeonju, Republic of Korea)	0.50	−1.33	2.23	−3.36	Nursery pigs	[113]
				1.00	1.56	0.48	1.12		

**Table 4:** Composition of crude protein (CP) and amino acids (AA-to-lysine ratio) of conventional protein supplements and bacterial protein supplements.

		Conventional Protein Supplement						Bacteria					
		Fish Meal		Soybean Meal		Blood Plasma		*Corynebacterium glutamicum*		*Methylophilus methylotrophus*		*Methylococcus capsulatus*	
CP, %		63.0		48.0		78.0		76.8		79.9		68.0	
Essential AA, %	Arg	3.80	83%	3.50	117%	4.40	64%	4.09	61%	3.61	80%	4.56	123%
	His	1.40	30%	1.30	43%	2.50	36%	1.55	23%	1.54	34%	1.54	42%
	Ile	2.60	57%	2.10	70%	2.70	39%	3.35	50%	3.32	73%	3.01	81%
	Leu	4.50	98%	3.60	120%	0.40	6%	5.38	80%	5.45	120%	5.06	137%
	Lys	4.60	-	3.00	-	6.90	-	6.74	-	4.54	-	3.70	-
	Met	1.70	37%	0.70	23%	0.80	12%	1.26	19%	1.83	40%	1.72	46%
	Phe	2.50	54%	2.40	80%	4.30	62%	2.78	41%	4.22	93%	2.70	73%
	Thr	2.60	57%	1.90	63%	4.50	65%	3.32	49%	3.97	87%	2.87	77%
	Trp	0.60	13%	0.70	23%	1.40	20%	0.56	8%	0.77	17%	2.21	60%
	Val	3.10	67%	2.20	73%	5.10	74%	4.61	68%	4.91	108%	3.94	106%
Non-essential AA, %	Ala	3.90	85%	2.10	70%	4.00	58%	6.26	93%	6.04	133%	4.64	125%
	Asp	5.40	117%	5.40	180%	7.40	107%	6.68	99%	7.52	166%	5.66	153%
	Cys	0.60	13%	0.70	23%	2.60	38%	0.35	5%	0.57	13%	0.45	12%
	Glu	7.90	172%	8.50	283%	10.90	158%	8.86	131%	10.50	231%	7.35	198%
	Gly	4.70	102%	2.00	67%	2.80	41%	3.33	49%	5.69	125%	3.34	90%
	Pro	2.90	63%	2.50	83%	4.30	62%	2.32	34%	NA	NA	2.57	69%
	Ser	2.40	52%	2.40	80%	4.20	61%	2.34	35%	2.60	57%	2.37	64%
	Tyr	1.90	41%	1.60	53%	3.90	57%	1.81	27%	3.48	77%	2.43	66%
Reference		[137]		[137]		[137]		[138,139]		[140,141]		[135,142,143]	

**Table 5:** Single-cell proteins from bacteria and their impacts on growth performance.

Micro-organism	Species	Product	Level, %	Improvement, %			Animal Model	Reference
				ADG	ADFI	FE		
Bacteria	*Corynebacterium ammoniagenes*	Protide, CJ	1.00	3.69	1.82	2.08	Broilers	[132]
			3.00	−1.02	−1.18	0.00		
			5.00	−3.07	−1.50	−1.56		
Bacteria	*Corynebacterium glutamicum*	Bacteria and medium	2.50	−4.32	−1.89	−2.08	Nursery pigs	[138]
			5.00	−8.38	1.89	−10.4		
Bacteria	*Corynebacterium glutamicum*	Lysed bacteria	0.70	−1.27	0.00	−3.64	Nursery pigs	[139]
			1.40	−1.27	5.31	−9.09		
			2.10	11.4	8.78	0.00		
Bacteria	*Methylococcus capsulatus*	BP, Dansk Bioprotein	10.7	5.59	1.31	3.44	Growing pigs	[131]
			12.0	8.48	7.82	−1.26	Nursery pigs	
			8.00	5.65	2.42	−2.52		
			4.00	−0.22	−4.55	3.77		
Bacteria	*Methylococcus capsulatus*	BBP, Norferm AS	6.00	2.68	−3.08	5.88	Broilers	[142]
			4.00	5.01	−0.57	5.29		
			2.00	3.98	0.81	3.53		
Bacteria	*Methylophilus methylotrophus*	Bacteria and medium	10.0	−1.44	0.20	1.78	Nursery pigs	[154]
			20.0	9.41	-0.08	1.78		
Bacteria	*Methylophilus methylotrophus*	Bacteria and medium	9.60	4.01	2.23	9.60	Broilers	[140]
			19.2	−14.2	−6.95	−8.09		
Bacteria	*Methylophilus methylotrophus*	Bacteria and medium	3.65	0.15	−4.00	−4.00	Broilers	[149]
			6.35	−1.00	−4.00	−3.00		
			9.00	−8.00	−8.00	−1.00		
			13.6	−13.0	−13.0	0.00		

**Table 6:** Composition of crude protein (CP) and amino acids (AA-to-lysine ratio) of single-cell protein from yeasts and microalgae.

		Yeast						Microalgae					
		Torula Yeast		*Saccharomyces cerevisiae*		*Yarrowia lipolytica*		*Desmodesmus* sp.		*Chlorella* sp.		*Nannochloropsis oceanica*	
CP, %		49.1		44.2		43.5		31.2		47.7		38.2	
Essential AA, %	Arg	2.39	72%	2.29	73%	1.81	55%	1.50	94%	3.88	123%	1.99	88%
	His	0.89	27%	1.05	34%	0.95	29%	0.50	31%	0.92	29%	0.64	28%
	Ile	2.16	66%	1.92	62%	1.99	61%	1.10	69%	1.87	60%	1.50	66%
	Leu	3.16	96%	2.99	96%	3.10	94%	2.30	144%	3.58	114%	2.90	128%
	Lys	3.30	-	3.11	-	3.28	-	1.60	-	3.15	100%	2.27	-
	Met	0.58	18%	0.73	23%	0.72	22%	0.50	31%	0.84	27%	0.57	25%
	Phe	1.92	58%	1.82	58%	1.54	47%	1.30	81%	2.12	67%	1.57	69%
	Thr	2.10	64%	2.19	70%	2.01	61%	1.30	81%	2.63	84%	1.54	68%
	Trp	0.59	18%	0.57	18%	0.65	20%	0.40	25%	0.24	7%	0.49	22%
	Val	2.49	76%	2.24	72%	2.39	73%	1.60	100%	3.44	109%	2.13	94%
Non-essential AA, %	Ala	3.03	92%	2.68	86%	3.63	111%	2.30	144%	1.39	44%	2.22	98%
	Asp	3.98	121%	4.49	144%	3.58	109%	2.70	169%	0.03	1%	2.80	123%
	Cys	0.46	14%	0.54	17%	0.44	13%	0.30	19%	0.42	13%	0.30	13%
	Glu	6.77	205%	6.57	211%	6.07	185%	2.90	181%	2.04	65%	3.34	147%
	Gly	1.94	59%	1.75	56%	1.96	60%	1.70	106%	2.43	77%	1.92	85%
	Pro	1.55	47%	2.10	68%	1.72	52%	2.70	169%	0.94	30%	4.00	176%
	Ser	1.78	54%	2.32	75%	1.82	55%	1.10	69%	0.78	25%	1.21	53%
	Tyr	1.48	45%	1.56	50%	1.50	46%	1.00	63%	1.77	56%	1.20	53%
References		[137,156,163,164]		[137]		[165]		[134]		[166,167,168]		[169]	

**Table 7:** Single-cell proteins from yeasts and their impacts on growth performance.

Micro-organism	Species	Product	Level, %	Improvement, %			Animal Model	Reference
				ADG	ADFI	FE		
Yeast	*Saccharomyces cerevisiae*	Autolyzed yeast	1.25	−1.05	5.56	1.89	Broilers	[136]
			2.50	−8.99	−0.20	−0.47		
			5.00	−12.6	2.69	−7.55		
Yeast	*Saccharomyces cerevisiae*	Whole yeast	0.50	4.32	−0.33	5.68	Broilers	[107]
		Yeast extract	0.30	3.74	2.45	2.84		
Yeast	*Saccharomyces cerevisiae*	Yeast and medium	3.00	−3.61	1.60	−2.27	Nursery pigs	[170]
Yeast	Torula yeast	Extracted yeast	4.00	−1.00	−3.00	−3.00	Broilers	[149]
			7.00	−3.00	−3.00	−1.00		
			10.0	−4.00	−3.00	1.00		
			15.0	−6.00	−5.00	1.00		
Yeast	Torula yeast	Yeast and medium	20.0	−4.87	−0.77	−4.44	Broilers	[163]
			4.75	1.38	−3.72	5.31		
Yeast	Torula yeast	SylPro, Arbiom Inc	10.8	8.76	2.29	6.76	Nursery pigs	[164]
			9.00	−3.75	−2.93	2.38		
			16.0	−3.00	−7.32	7.77		
			23.0	−7.87	−12.4	2.69		
Yeast	*Yarrowia lipolytica*	Yeast and medium	3.00	12.4	−1.25	11.9	Nursery pigs	[165]
			6.00	−1.81	−1.63	−0.63		
Yeast	*Yarrowia lipolytica*	Yeast and medium	3.00	2.27	−1.20	2.14	Nursery pigs	[170]

**Table 8:** Single-cell proteins from microalgae and their impacts on growth performance.

Micro-organism	Species	Product	Level, %	Improvement, %			Animal Model	Reference
				ADG	ADFI	FE		
Microalgae	*Arthrospira platensis*	Spirulina, Sopropeche	15.0	−11.5	−2.42	−10.1	Broilers	[174]
Microalgae	*Arthrospira platensis*	Spirulina powder, Sopropeche	10.0	−12.4	−3.71	−9.46	Nursery pigs	[180]
Microalgae	*Chlorella* sp.	Pure, whole	7.50	0.21	2.32	−2.50	Broilers	[166]
			15.0	−2.07	−0.51	−1.87		
Microalgae	*Chlorella* sp.	Allmicroalgae, Natural Products	5.00	12.6	4.30	7.43	Finishing pigs	[167]
Microalgae	*Chlorella* sp.	Allmicroalgae, Natural Products	10.0	5.44	−3.48	3.14	Broilers	[168]
Microalgae	*Desmodesmus* sp.	DGM, Cellana	10.0	−11.4	−9.73	−1.64	Nursery pigs	[134]
			15.0	5.21	8.56	16.42	Broilers	
Microalgae	*Desmodesmus* sp.	Pure, whole	5.00	35.36	−0.24	12.24	Broilers	[179]
		Pure, defatted	5.00	20.91	−2.37	5.44		
Microalgae	*Nannochloropsis oceanica*	DGA, Cellana	2.00	0.84	−1.67	1.54	Broilers	[169]
			4.00	−2.39	0.83	−3.08		
			8.00	1.97	3.33	0.00		
			16.0	−10.4	−3.33	−6.15		
Microalgae	*Staurosira* sp.	Pure, defatted	7.50	1.20	6.21	−4.78	Broilers	[51]

**Table 9:** Fatty acid composition (% of total lipids) in different sources of lipid supplements.

	Poultry Fat	Soybean Oil	*Yarrowia lipolytica*	*Schizochytrium* sp.	*Crypthecodinium cohnii*
ME, kcal/kg	8364	8574	-	-	-
Total saturated, %	28.7	14.2	19.4	36.5	-
Total unsaturated, %	64.8	81.0	80.6	62.4	-
FA, %				-	
C 14:0	0.9	0.1	0.3	11.0	16.0
C 16:0	21.6	10.3	10.7	38.5	25.0
C 16:1	5.7	0.2	1.5	18.5	0.4
C 18:0	6.0	3.8	6.6	1.10	-
C 18:1	37.4	22.8	8.8	3.15	16.0
C 18:2	19.5	51.0	22.9	-	0.5
C 18:3	1.0	6.8	2.3	-	0.4
C 20:0	-	-	0.7	-	-
C 20:1	1.1	0.2	0.2	0.60	-
C 20:4	0.1	0.0	4.0	-	-
C 20:5	0.0	0.0	30.2	1.65	0.1
C 22:1	0.0	0.0	0.9	0.10	-
C 22:5	0.0	0.0	0.9	12.9	-
C 22:6	0.0	0.0	-	24.0	39.0
References	[137]	[137]	[206]	[208,209]	[207]

**Table 10:** Single-cell oils from microorganisms and their impacts on growth performance.

Microorganism	Species	Level, %	Improvement, %			Animal Model	Reference
			ADG	ADFI	FE		
Yeast	*Yarrowia lipolytica*	1.50	15.2	−5.08	20.0	Nursery pigs	[213]
		3.00	4.64	−3.23	7.27		
Microalgae	*Aurantiochytrium acetophilum*	1.00	−2.65	6.42	−8.18	Broilers	[215]
		2.00	−4.05	1.83	−6.21		
		4.00	−12.7	1.83	−14.7		
Microalgae	*Schizochytrium* sp.	3.12	−0.23	−3.69	4.35	Nursery pigs	[216]
Microalgae	*Schizochytrium* sp.	3.60	−1.92	−0.95	−0.86	Growing-finishing pigs	[217]
Microalgae	*Schizochytrium* sp.	3.70	−0.93	−3.03	3.45	Growing-finishing pigs	[218]
Microalgae	*Schizochytrium* sp.	0.25	4.17	0.65	3.38	Growing-finishing pigs	[116]
		0.50	4.17	1.18	3.80		

## Data Availability

Not applicable.
